# Regulation of growth, invasion and metabolism of breast ductal carcinoma through CCL2/CCR2 signaling interactions with MET receptor tyrosine kinases

**DOI:** 10.1016/j.neo.2022.100791

**Published:** 2022-04-08

**Authors:** Diana Sofía Acevedo, Wei Bin Fang, Vinamratha Rao, Vedha Penmetcha, Hannah Leyva, Gabriela Acosta, Paige Cote, Rebecca Brodine, Russell Swerdlow, Lin Tan, Philip L Lorenzi, Nikki Cheng

**Affiliations:** a Department of Pathology and Laboratory Medicine, University of Kansas Medical Center, 3901 Rainbow Boulevard, Wahl Hall East 1020, Kansas City, KS 66160, USA; b Department of Cancer Biology, University of Kansas Medical Center, Kansas City, KS 66160, USA; c Department of Neurology, University of Kansas Medical Center, Kansas City, KS 66160, USA; d Department of Bioinformatics and Computational Biology, The University of Texas MD Anderson Cancer Center, Houston, TX 77030, USA

**Keywords:** CCL2, CCR2 GPCR, MET receptor tyrosine kinase, LY2801653, Metabolism, Breast ductal carcinoma, 2-DG, 2-deoxyglucose, a-sma, alpha smooth muscle actin, ANOVA, analysis of variance, CRISPR, clustered regularly interspaced short palindromic repeats, CK19, cytokeratin 19, DCIS-mi, Ductal carcinoma *in situ* with microinvasion, ECAR, extracellular acidification rate, gRNA, guide RNA, GPCR, G coupled protein receptor, HK2, hexokinase 2, IC-MS, Ion Chromatography Mass Spectrometry, IDC, invasion ductal carcinoma, KUMC, University of Kansas Medical Center, LDH, lactate dehydrogenase, MET-KO, MET knockout, MIND, Mammary Intraductal, NCI, National Cancer Institute, NOD-SCID, Non-Obese Diabetic Severe Combined Immunodeficient, OCR, oxygen consumption rate, PCNA, proliferating cell nuclear antigen, PKM, pyruvate kinase M, PLA, proximity ligation assay, RTK, receptor tyrosine kinase, TCGA, The Cancer Genome Atlas

## Abstract

With over 60,000 cases diagnosed annually in the US, ductal carcinoma *in situ* (DCIS) is the most prevalent form of early-stage breast cancer. Because many DCIS cases never progress to invasive ductal carcinomas (IDC), overtreatment remains a significant problem. Up to 20% patients experience disease recurrence, indicating that standard treatments do not effectively treat DCIS for a subset of patients. By understanding the mechanisms of DCIS progression, we can develop new treatment strategies better tailored to patients. The chemokine CCL2 and its receptor CCR2 are known to regulate macrophage recruitment during inflammation and cancer progression. Recent studies indicate that increased CCL2/CCR2 signaling in breast epithelial cells enhance formation of IDC. Here, we characterized the molecular mechanisms important for CCL2/CCR2-mediated DCIS progression. Phospho-protein array profiling revealed that CCL2 stimulated phosphorylation of MET receptor tyrosine kinases in breast cancer cells. Co-immunoprecipitation and proximity ligation assays demonstrated that CCL2-induced MET activity depended on interactions with CCR2 and SRC. Extracellular flux analysis and biochemical assays revealed that CCL2/CCR2 signaling in breast cancer cells enhanced glycolytic enzyme expression and activity. CRISPR knockout and pharmacologic inhibition of MET revealed that CCL2/CCR2-induced breast cancer cell proliferation, survival, migration and glycolysis through MET-dependent mechanisms. In animals, MET inhibitors blocked CCR2-mediated DCIS progression and metabolism. CCR2 and MET were significantly co-expressed in patient DCIS and IDC tissues. In summary, MET receptor activity is an important mechanism for CCL2/CCR2-mediated progression and metabolism of early-stage breast cancer, with important clinical implications.

## Highlights

• CCR2 correlates with MET receptor expression in breast ductal carcinomas.
• CCL2/CCR2 signaling in breast cancer cells depend on interactions with MET.
• CCR2 and MET signals alter metabolism of ductal carcinoma *in situ* in animal models.
• CCR2 mediates metabolism and progression of MIND lesions through MET.

## Introduction

With over 60,000 cases diagnosed annually in the US, ductal carcinoma *in situ* (DCIS) is the most prevalent form of early-stage breast cancer. Considered the immediate precursor to invasive breast ductal carcinomas (IDC), DCIS is characterized by the growth of neoplastic cells within the breast ducts. Standard treatment for DCIS involves surgery and radiotherapy, with or without adjuvant endocrine therapy [1]. Because many DCIS cases may never progress to IDC, overtreatment remains a significant problem, reducing the quality of life for patients [2]. Yet, up to 20% patients experience disease recurrence, with half of cases presenting invasive disease, indicating that standard treatments do not effectively treat DCIS for a subset of patients [1]. Currently, there are no reliable approaches to determine which DCIS cases will become invasive. Small or low-grade DCIS lesions may still become invasive [3]. Molecular subtype of breast cancer is important in determining prognosis of IDC [4] but has less prognostic value for DCIS [5]. Increased expression of biomarkers: COX-2, FOXA1, HER2, Ki-67, p16/INK4A, PR, and SIAH2 is associated with increased risk of recurrence in some DCIS cases [6,7]. By understanding the underlying mechanisms of DCIS progression, we can identify new biomarkers associated with development of IDC and develop treatment strategies better tailored to the patient.

Chemokines are small soluble molecules (∼8 kda) that regulate the homing and trafficking of immune cells during wound healing, infection and cancer progression. Chemokines bind to G protein coupled receptors to regulate cell cellular adhesion, proliferation, migration, and expression of inflammatory mediators. The chemokine family is subdivided into several classes (C-C, CXC, CXC3C or XC), based on the composition of a conserved cysteine motif. C-C chemokines are defined by their roles in regulating T cell and macrophage activity. The C-C Ligand 2 (CCL2) and its primary receptor CCR2 are key regulators of macrophage recruitment, and their expression are upregulated in prostate, glioma and breast cancers [8]. Though CCL2/CCR2 signaling is known to promote late-stage tumor growth and metastasis by enhancing macrophage recruitment to the primary tumor [9,10], CCL2 increases tumor cell growth, survival and migration by signaling to CCR2 overexpressing breast carcinoma cells [11]. Increased expression of CCR2 in DCIS tissues indicate that CCL2/CCR2 signaling occurs in early-stage disease [12].

Mammary fat pad and subcutaneous injection of human cells in mice are commonly used to model breast tumors, but do not accurately replicate the growth and progression of human DCIS. In contrast, mammary intra-ductal (MIND) injection of breast cancer cells leads to formation of DCIS lesions that eventually escape myoepithelial barriers and invade into the surrounding stroma, resulting in IDC. The MIND model mimics progression of human DCIS more closely than conventional transplant models [13,14]. In recent studies, MIND injection of transformed and patient derived breast cancer cells resulted in DCIS, which became invasive with CCL2 treatment [12]. CCR2 knockdown in DCIS.com breast cancer cells inhibited the growth and invasion of breast lesions in the MIND model. CCR2 overexpression in lowly invasive SUM225 breast cancer cells enhanced their growth and invasion *in vivo* [15]. Thus, increased CCL2 levels and CCR2 overexpression in breast epithelial cells enhance progression of DCIS lesions. However, it remains unclear how CCL2/CCR2 signaling regulates DCIS progression, in the context of other oncogenes.

Tissue homeostasis requires the balance of energy production and consumption of nutrients such as glucose. In normal mammalian cells, glucose is metabolized into pyruvate, which is converted into acetyl COA, fueling the TCA cycle and oxidative phosphorylation for the generation of ATP. Glycolysis and the TCA cycle provides building blocks for synthesis of lipids, amino acids and nucleotides. In cancer cells, glucose metabolism becomes reprogrammed to generate lactate, in a process known as anaerobic glycolysis (Warburg effect). Anaerobic glycolysis provides a growth advantage to late-stage breast cancers and contributes to tamoxifen resistance in hormone receptor positive breast cancers through lactate, AKT, and c-MYC-dependent mechanisms [16], [17], [18]. Metabolic reprogramming is a hallmark of IDC but remains poorly understood in DCIS. Glycolytic biomarker expression is upregulated in DCIS and IDC [19,20], suggesting that metabolic changes may occur during DCIS progression. A recent study showed that CCL2 overexpression affected mitochondrial function, including oxidative phosphorylation in liver and muscle tissues [21], suggesting a link between CCL2/CCR2 signaling and metabolism.

Here, we sought to understand the molecular mechanisms through which CCL2/CCR2 signaling regulated DCIS progression using *in vitro* and *in vivo* breast cancer models. We demonstrate that CCR2 mediates breast cancer growth, invasion and glucose metabolism through MET receptor-dependent mechanisms. Furthermore, we demonstrate a clinical relevance for CCR2 and MET co-expression in breast tissues, with important implications for treatment.

## Materials and methods

### Cell culture

DCIS.com and MCF7 cells were cultured as described [22,23]. HCC1937 cells [24] were cultured in RPMI/10% FBS, 2mM L-glutamine/1% penicillin-streptomycin. Control or CCR2-overexpressing SUM225 cells were generated and cultured as described [15]. Cell lines were cultured for less than 6 months at a time and analyzed after thawing using the MycoAlert™ Mycoplasm Detection Kit (Lonza, cat no.LT07–703).

### Reagents

Recombinant human HGF (cat no.100-39H) and human CCL2 (cat no.300-04) are from Peprotech. PP2 are from Tocris (cat no.1407). The Eli Lily stock of MET inhibitor LY2801653 was obtained from Lot no. KW1-E02099-043-A. For *in vitro* studies, LY2801653 was resuspended in DMSO. IC50 assays were conducted to determine optimal concentrations. Unless otherwise stated, *in vitro* experiments were conducted using: 100 ng/ml recombinant protein, 10 mM PP2 and/or 203 nM LY2801653 in serum free media. For *in vivo* studies, LY2801653 was solubilized in 20% Captisol (Cydex Pharmaceuticals) in water and formulated in 10% PEG 400/90% as described [25].

### Proximity ligation assay

Protein interactions were assessed using the Duolink PLA assay (Sigma). Briefly, cells were fixed with 10% neutral buffer formalin, permeabilized with PBS/0.2% Triton X-100 and incubated with the following antibody pairs: anti-CCR2 (1:100 dilution, Biolegend, cat no.357202) and anti-SRC (1:100, Cell Signaling Technology, cat no.2123S), anti-MET (1:50, Santa Cruz Biotechnology, cat no.SC-514148) and anti-SRC, or anti-CCR2 and anti-MET (1:100, Cell Signaling Technology, cat no.4560). Samples were incubated with Duolink *in situ* Probes; anti-rabbit Minus (cat no.DUO92005) and anti-mouse PLUS (cat no.DUO92001). Signals were amplified with polymerase using *In Situ* Detection Reagents Green (cat no.DUO92014). Duolink PLA wash buffers (cat no.DUO82049) were used. Cells were counterstained with DAPI. Images were captured at 10x magnification using the FL-Auto EVOS imager.

### Wound closure

Confluent monolayers of cells were grown in 24 well plates, serum starved for 24 h and scratched with/without recombinant protein. Four images/sample were captured at 0 and 24 h at 10x magnification using the FL-Auto EVOS imager. Wound closure was assessed by ImageJ as described [11].

### Flow cytometry

500,000 cells were detached with Accutase (EMD Millipore, Cat no.SCR005). Cells were incubated for 30 min in PBS with anti-MET-FITC (0.1 mg/ml, SinoBiological, cat no.10692-R243-F) and/or anti-CCR2-PE (5ml/1million cells; Biolegend, cat no.357205). Cells were analyzed using a BD LSRII Flow Cytometer and normalized to respective unstained controls.

*Spheroid growth assay*

Cells were cultured in Collagen:Matrigel matrix using procedures previously described [26]. Briefly, rat tail collagen was mixed with 1:1 with Growth Factor Reduced Matrigel (BD Biosciences, cat no.354230) and coated on to 96-well plates. Plates were incubated with 2500 breast cancer cells/well in 200ml DMEM/10% FBS/2.5% Matrigel for 10 days. 4 fields/well at 10x magnification were captured using the FL-Auto EVOS imager. Sphere size was quantified using ImageJ and normalized to the total number of spheroids.

### Immunofluorescence on cultured cells

60,000 cells/well in 24 well plates were fixed with 10% neutral formalin buffer, permeabilized with methanol and blocked with PBS/3% FBS. Cells were immunostained with antibodies (1:500) to: PCNA (BioLegend, cat no.307902) or cleaved caspase-3 (Asp-175; Cell Signaling Technologies, cat no.9661). PCNA was detected using secondary donkey anti-mouse Alexa-Fluor568 (Invitrogen, cat no.A10037). Cleaved caspase-3 was detected using secondary donkey anti-rabbit Alexa Fluor647 (Invitrogen, cat no.A-31573). Samples were counterstained with DAPI in 50% glycerol/PBS. Four images/well were captured at 10x magnification using the FL-Auto EVOS imager.

### CRISPR gene ablation

MET gRNA (BRDN0001147560) [27] was a gift from Dr. John Doench and David Root (Addgene plasmid no.76061; http://n2t.net/addgene:76061). It encodes for the IPT domain, 808 to 827. EGFP gRNA (BRDN0000561167) [27] was a gift from Drs. Doench and Root (Addgene plasmid # 80034; http://n2t.net/addgene:80034). pLenti-Cas9 was a gift from Dr. Jeremy Chien (UC-Davis). Cells were transduced with plenti-cas9 and selected with Blasticidin (10 mg/ml). Cas9 positive cells were then transduced with lentivirus carrying gRNAs and selected with puromycin (5mg/ml).

### Extracellular flux analysis

30,000 cells/well were seeded in 24-well plates, serum starved, and treated with/without recombinant protein, with six replicates/group. XF Glycolysis stress tests (Agilent cat no.100850-001) were performed according to manufacturer's instructions. Measurements were obtained after injection of: 10nM glucose, 1µM oligomycin, 100mM 2-deoxyglucose (2D-G) using the XF24 extracellular flux analyzer (Seahorse Bioscience). Three readings/experiment were taken to ensure stability.

### Glycolytic enzyme assay

100,00 cells/well were seeded in 24 well plates, serum starved for 24 h and treated with/without recombinant protein for up to 24 h. Enzyme activity was determined by Hexokinase/Glucokinase assay (Biomedical Research Service, cat no.E-111) or Pyruvate Kinase assay (Biomedical Research Service, cat no.E-117).

### Glucose consumption/lactate assay

60,000 cells/well were seeded in 24 well plates, serum starved for 24 h, and treated with/without recombinant protein or LY2801653 for up to 48 h. Cells or media were assayed using the Glucose-Glow™ Assay (Promega, cat no.J6021) or Lactate-Glo™ Assay (Promega, cat no.J5021).

### Co-immunoprecipitation/immunoblot

For co-immunoprecipitation, 2 million cells/10 cm dish were serum starved for 24 h, and treated with/without recombinant protein for up to 30 min. Cells were lysed in RIPA buffer with protease inhibitors (Gendepot, cat no.3100-005) and phosphatase inhibitors (Gendepot, cat no.P3200-005). 1mg/500ml lysate were precleared with 2mg mouse IgG (Sigma, cat no.I-5381) or rabbit IgG (Sigma, cat.#I-5006), and 100 ml A/G beads (Santa Cruz Biotechnology, cat no.SC-2003). Samples were immunoprecipitated with 2mg IgG control or antibodies to MET (Santa Cruz Biotechnology, cat no.SC-514148) or SRC (Cell Signaling Tech, cat no.2123S) bound to A/G beads, washed in RIPA buffer, denatured in loading buffer and resolved by SDS-PAGE.

For immunoblot, 100,000 cells/well in 24 well plates were lysed in RIPA buffer with protease/phosphatase inhibitors. 30mg protein was resolved on SDS-PAGE.

Nitrocellulose membranes were immunoblotted with anti-CCR2-HRP (1:200, Santa Cruz Biotechnology, cat no.SC74490HRP) or with antibodies (1:1000) to: phospho-SRC (Y416) (Cell Signaling Technology, cat no.6943S), SRC, pY1234/1235-MET (Cell Signaling Technology, cat no.3077S), pY1349-MET (Cell Signaling Technology, cat no.3121S), MET, Hexokinase II (HK2) (Cell Signaling Technology, cat no.2867), PKM1/2 (Cell Signaling Technology, cat no.3190S), Lactate Dehydrogenase A-D (Santa Cruz Biotechnology, cat no.sc-133123), or β-actin (Sigma, cat no.A5441). Proteins were detected using appropriate secondary antibodies. Membranes were developed with West Pico ECL chemiluminescent substrate and imaged using a Biospectrum Imaging System. Densitometry was performed using Image J.

### MIND model

Non-Obese Diabetic Severe Combined Immunodeficient interleukin receptor-γ2 null female mice (NOD-SCID), 8–10 weeks old, were purchased from Jackson Laboratories. MIND injections were performed using procedures described [13]. Briefly, animals were anesthetized with 100 mg/kg ketamine with injection of 10 mg/kg xylazine. #4/5 or #9/10 nipples were clipped. 20,000 cells (5µL) in PBS/0.1% trypan blue were injected through the nipples. Four weeks later, mice were dosed by oral gavage with vehicle control or 12 mg/Kg of LY2801653 once daily in a 5/2 schedule for 4 weeks. Mice were monitored twice a week until endpoint.

### Immunohistochemistry/co-immunofluorescence

Tissue processing, PCNA and cleaved caspase-3 immunostaining were performed using procedures described [28]. For CCR2 (Biolegend cat no.357201), MET (Cell Signaling Technology cat no.9579), HK2 and PKM1/2 staining, dewaxed five-micron sections were heated at low pressure in 2M urea pH 6.8 for 3 min. After quenching endogenous peroxidases with 60% methanol/3% H_2_0_2,_ slides were blocked in PBS/3% FBS and incubated with primary antibodies (1:100) overnight at 4°C. For DAB staining, sections were incubated with appropriate secondary biotinylated antibodies, then with streptavidin-peroxidase (Vector Laboratories, cat no.PK-6200). Proteins were detected using 3,3’-diaminobenzidine (DAB) (Vector Laboratories, cat no.SK-4100). Slides were counterstained with Mayer's hematoxylin and mounted with Cytoseal. For co-immunofluorescence, MET was detected by mouse biotinylated antibodies bound to streptavidin-alexa-488 (Invitrogen cat no.S11223). CCR2 was detected by anti-rabbit-alexa-Fluor568 (Invitrogen cat no. A-11011). Slides were counterstained with DAPI. 4 images at 10x magnification were captured using the FL-Auto EVOS imager and quantified by Image J as described [15].

### Assessment of invasion *in vivo*

Sections were co-immunofluorescence stained for Cytokeratin 19 (CK19) and α-smooth muscle actin (a-sma) using procedures previously described [15]. Ten images/slide were captured at 10x magnification and scored in a blinded manner. Scoring was defined as: 1= DCIS, with epithelial cells confined within a duct lined by a-sma+ myoepithelium, 2= DCIS+Microinvasion, (Mi) with ≤50% disruption of α-sma+ myoepithelium and < 3 cells invading through the duct, 3= IDC, with disappearance of > 50% α-sma and ≥3 invading cells contacting the stroma.

### Ion chromatography mass spectrometry (IC-MS)

Mammary tissues were harvested 24 h after the last dosage of LY2801653 and flash-frozen with liquid nitrogen. 10mg tissue were placed in a pre-weighted cryotube (Bertin, cat no.CK28-R). Samples were analyzed using ion chromatography mass spectroscopy (IC-MS) using procedures previously described [29].

## Dataset analysis

mRNA breast cancer TCGA datasets (*n* = 963) [30,31] were accessed on cbioportal.org on July 13,2021 to examine CCR2 and MET expression.

## Statistical analysis

Unless otherwise stated, *in vitro* experiments were conducted with triplicate samples and performed a minimum of three times. For animal experiments, sample size analysis was determined using PS Power and Sample Size Program Ver3.0, based off data on lesion mass. Minimum of *n = *8/group was needed to reach a sufficient power of 0.80 with alpha=0.05. Statistical analysis was performed using GraphPad Prism software. Kolmogorov–Smirnov test of normality distribution was performed. Spearman correlation tests were used for data with non-normal distribution. Student's two-tailed T test was used for two groups of data normally distributed. One-way ANOVA with Bonferroni's post-hoc comparison was used for >2 groups of data normally distributed. Statistical significance was determined as * *p* < 0.05, **p < 0.01, ***p < 0.001, ns=not significant.

### Patient specimens

For co-immunofluorescence, biospecimens of pure DCIS, DCIS co-occurring with IDC (Co-DCIS) and IDC (*n = *9/group), were obtained from the Cooperative Human Tissue Network (CHTN) and from the Biospecimen Repository Core Facility (BRCF) at the University of Kansas Medical Center (KUMC). For DAB staining, tissue microarrays were generated as previously described [12].

### Ethics statements on human tissues and animal subjects

Tissues were classified as “Exempted” and approved for study by the Human Research Protection Program at KUMC (#080193). The BRCF, a facility approved by the Institutional Review Board (IRB), obtained written informed consent for tissue collection. The CHTN is a network of six academic institutions with IRB-approved facilities, which collects and distributes remnant human biospecimens from routine surgeries and autopsies to biomedical researchers. All samples were de-identified prior to distribution. Existing medical records were used in compliance with KUMC and NCI regulations. These regulations are aligned with the World Medical Association Declaration of Helsinki. Animals were maintained at KUMC in accordance with the Association for Assessment and Accreditation of Laboratory Animal Care. Animal experiments were performed under a protocol approved by the KUMC Institutional Animal Care and Use Committee and complied with the ARRIVE guidelines.

## Results

### CCL2/CCR2 enhance MET phosphorylation through SRC-dependent mechanisms in breast cancer cells

To understand how CCL2/CCR2 signaled in breast cancer cells in the context of other oncogenic receptors, we used candidate and unbiased profiling approaches. Through analysis of data obtained from reverse phase protein profiling in a previous study [32], we found that CCL2 treatment of MCF10CA1d breast cancer cells enhanced phosphorylation of MET receptors, which was reduced in CCR2 knockout cells (Supplemental Fig. 1).

MET RTKs normally regulate ductal branching and outgrowth during mammary gland development [33,34]. MET is overexpressed in IDC, and regulates breast tumor growth, metastasis and chemoresistance. Hepatocyte Growth Factor (HGF) ligand binding triggers receptor dimerization and autophosphorylation of tyrosine residues Y1234/5 in the activation loop of the kinase domain, and Y1349 in the C-terminal tail, a docking site for downstream effectors that facilitate breast cancer cell survival, proliferation, invasion and scattering [35]. To date, a functional connection between CCR2 and MET in breast cancer has not been clearly identified.

**Fig. 1:**
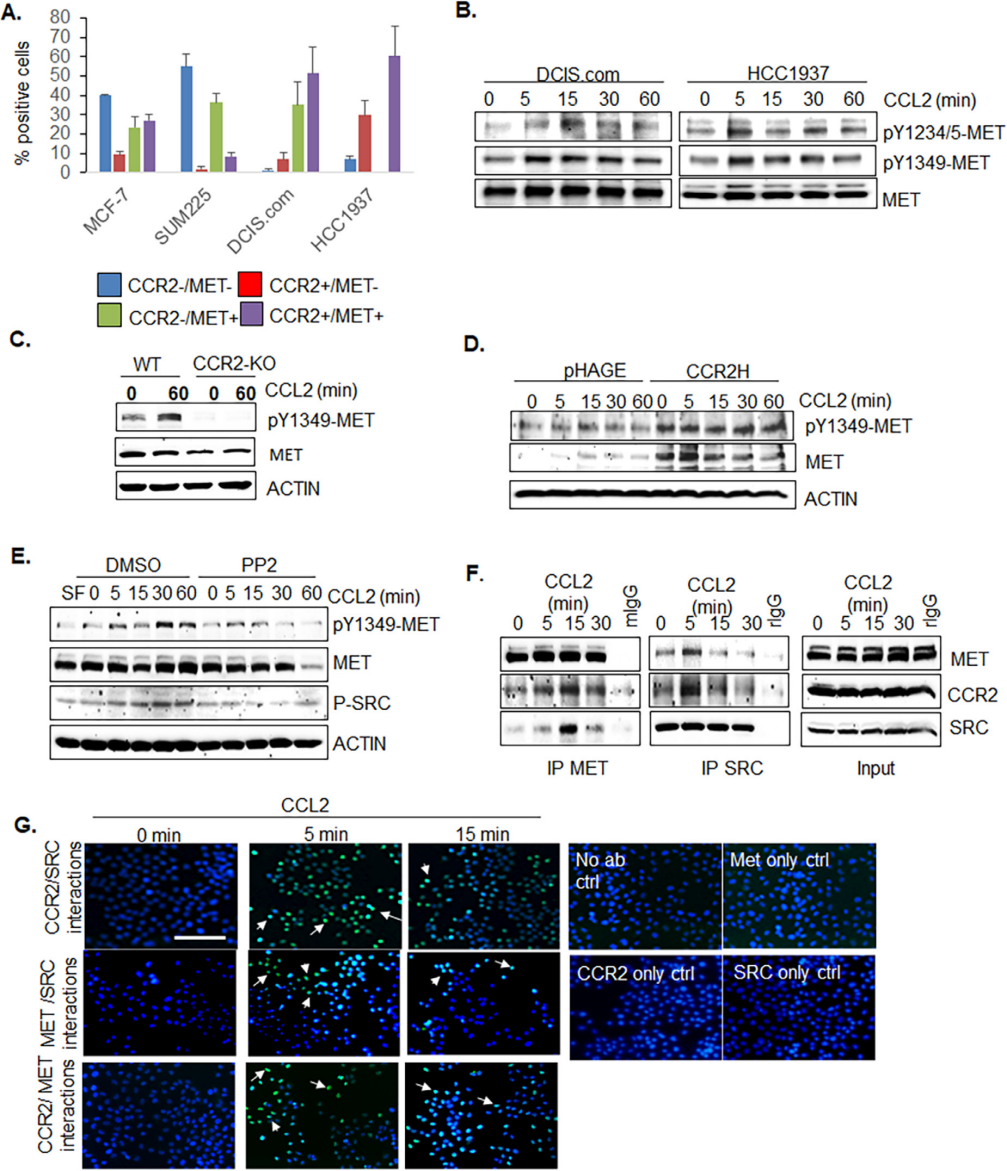
CCL2 stimulation and CCR2 expression enhance MET phosphorylation through SRC dependent mechanisms in breast cancer cells. A. Flow cytometry for CCR2/MET co-expression in breast cancer cell lines. B-E. Immunoblot analysis on the effects of CCL2 treatment (100 ng/ml) of DCIS.com and HCC1937 cells (B), DCIS.com with wildtype CCR2 (WT) or CRISPR CCR2 knockout (CCR2-KO) (C), SUM225 pHAGE control or CCR2 overexpressing cells (CCR2-H) (D), and DCIS.com breast cancer cells with DMSO control with/without CCL2 and/or 10 mM PP2 (E). F. Co-immunoprecipitation analysis of DCIS.com cells treated with CCL2 for up to 30 min. G. Proximity Ligation assay for DCIS.com breast cancer cells treated with CCL2 for 5 or 15 min. Arrows point to positive signals (green fluorescence spots). DAPI overlay. Scale bar=50 microns.

We profiled receptor expression in breast cancer cell lines by flow cytometry based on tumorigenicity. DCIS.com and HCC1937 cells transplanted in mice form rapidly breast carcinomas that become highly invasive [15,36]. SUM225 and MCF7 cells form slower growing, lowly invasive breast carcinomas in mice [15,37]. CCR2 and MET were co-expressed at higher levels in DCIS.com and HCC1937 cells, compared to MCF-7 and SUM225 cells (Fig. 1A). CCL2 treatment of DCIS.com and HCC1937 cells enhanced MET phosphorylation at Y1234/5 and Y1349 as early as 5 min. Notably, Y1349 phosphorylation lasted for up to 60 min (Fig. 1B). CCL2-induced MET phosphorylation was inhibited with CCR2 knockout in DCIS.com cells (Fig. 1C) and enhanced with CCR2 overexpression in SUM225 (CCR2-H) cells (Fig. 1D). These data indicate that CCL2 and CCR2 are important for MET phosphorylation in breast cancer cells.

To understand how CCL2 and CCR2 regulated MET phosphorylation, HGF levels were first examined. By ELISA, CCL2 treatment and CCR2-KO did not affect HGF expression in DCIS.com cells (Supplemental Fig. 2A,B). We then determined whether CCL2/CCR2 signaling regulated phosphorylation through a signaling intermediate. As CCL2/CCR2 activated SRC [32] and SRC interacts with MET to regulate receptor activation in carcinoma cells [38,39], we asked whether CCL2-mediated SRC activation was important for MET phosphorylation. SRC inhibition through PP2 treatment reduced CCL2-induced MET phosphorylation, corresponding to decreased SRC phosphorylation in DCIS.com cells (Fig. 1E). We then assessed for potential interactions among CCR2, MET and SRC in CCL2-treated cells. CCL2 treatment of DCIS.com cells resulted in co-immunoprecipitation of MET with CCR2 and SRC, and SRC with MET and CCR2 (Fig. 1F). As a complementary approach, protein interactions were examined by proximity ligation assay. We detected increased interactions between MET and SRC and CCR2 and MET, and interactions between CCR2 and SRC at 5 and 15 min. The strongest protein interactions appeared at 5 min (Fig. 1G). Overall, these data indicate that: CCL2 triggers interactions among CCR2, MET and SRC in breast cancer cells, and CCL2 regulates MET phosphorylation through CCR2- and SRC-dependent mechanisms.

### CCL2 regulates breast cancer cell migration, proliferation, survival and metabolism through MET-dependent mechanisms

**Fig. 2:**
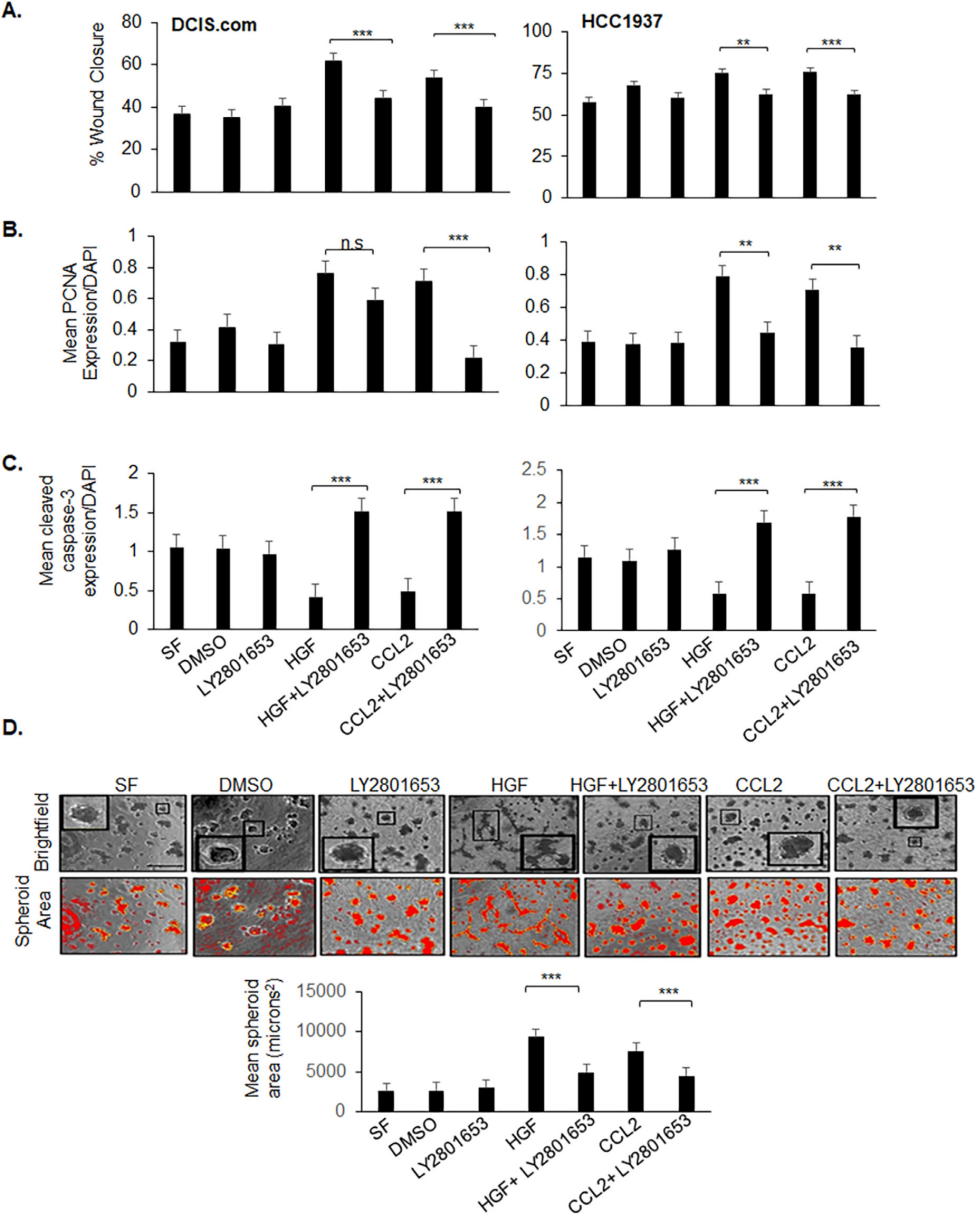
LY2801653 blocks CCL2-induced cell migration, proliferation, and survival in breast cancer cells. DCIS.com or HCC1937 breast cancer cell lines were treated in serum free (SF) media with/without: 100 ng/ml HGF (positive control), 100 ng/ml CCL2, 203 nM LY2801653 or DMSO vehicle control. Cells were analyzed for changes in A. wound closure, B. cell proliferation through PCNA immunostaining, and C. cell death by cleaved caspase-3 immunofstaining. D. DCIS.com cells were measured for changes in spheroid growth in Matrigel:Collagen after 10 days in culture. Spheroid growth and biomarker expression were measured by Image J. Statistical analysis was performed using a One Way ANOVA with Bonferroni post-hoc comparison. Statistical significance was determined by *p < 0.05, **p < 0.01, ***p <  0.0001., n.s. not significant. Mean ± SEM are shown. Scale bar=200 microns.

To determine the functional relevance of CCL2-mediated MET phosphorylation, we examined the effects of inhibiting MET activity in DCIS.com and HCC1937 cells, which co-expressed CCR2 and MET at high levels. Cells were treated with LY2801653 (Merestinib), a type II ATP competitive inhibitor, or subject to CRISPR knockout of MET (MET-KO). In DCIS.com and HCC1937 cell cultures, CCL2 enhanced wound closure, proliferation, and survival to similar levels as HGF. LY2801653 treatment or MET-KO inhibited CCL2- and HGF-induced wound closure, cell proliferation, survival and spheroid growth (Fig. 2A–D, Supplemental Fig. 3A–D). Overall, CCL2 enhances breast cancer cell migration, proliferation, and survival through MET-dependent mechanisms.

**Fig. 3:**
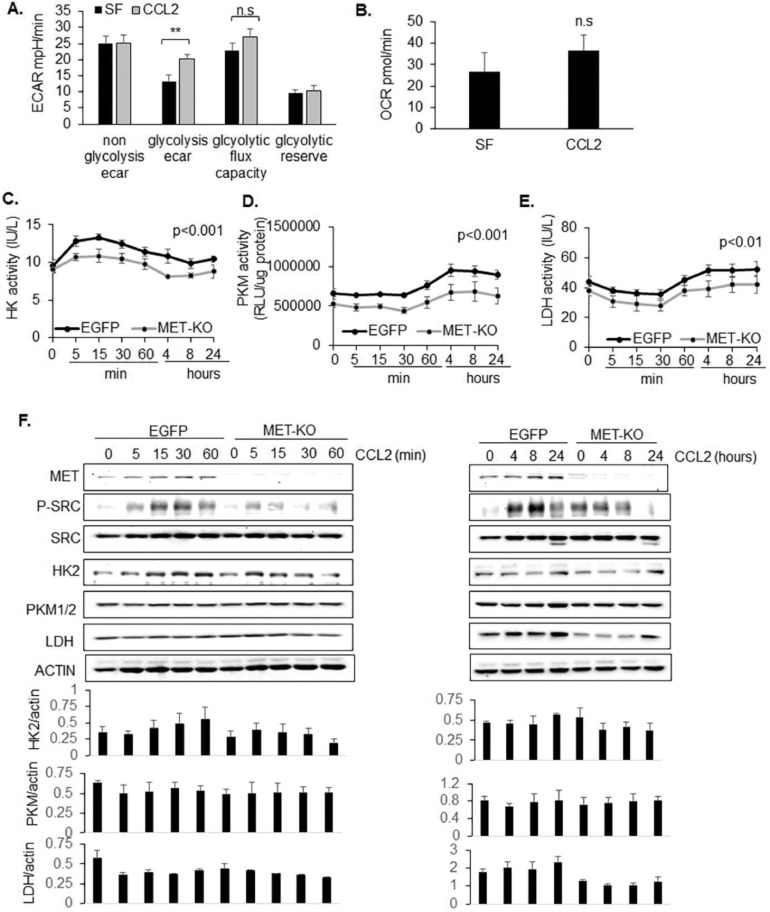
MET-KO inhibits CCL2 mediated glycolysis in breast cancer cells A-B. DCIS.com cells were treated with/without 100 ng/ml CCL2, subject to glycolysis stress tests, and analyzed for changes in extracellular acidification rate (ECAR) (A) and oxygen consumption rate (OCR) (B) using the Seahorse metabolic flux analyzer. C-F. DCIS.com EGFP control or MET-KO cells were treated with/without CCL2 for up to 24 h and analyzed for changes in activity: of Hexokinase (C), Pyruvate kinase M (D) or Lactate dehydrogenase (LDH) (E), or expression of indicated proteins by immunoblot (F). Densitometry of immunoblots was performed using Image J. Statistical analysis was determined by One-Way ANOVA with Bonferroni post hoc test. Statistical significance was determined by *p < *0.05. *p < 0.05. Mean+SEM are shown.

Previously, we demonstrated that CCR2-KO in breast cancer cells inhibited tumor growth [32]. It also increased the pH in phenol red containing media, indicating potential changes in bioenergetics. We first examined effects of CCL2 on metabolism in DCIS.com and HCC1937 cells, which expressed high levels of endogenous CCR2. CCL2 treatment enhanced glucose consumption in both cell lines. The functional contribution of CCR2 to metabolism was then assessed in cells with stable CCR2 overexpression. CCL2 treatment enhanced glucose consumption in SUM225 CCR2-H cells compared to pHAGE controls (Supplemental Fig. 4A,B). Through glycolysis stress tests in the Seahorse extracellular flux analyzer, CCL2 treatment was found to enhance the rate of extracellular acidification but not oxygen consumption in DCIS.com cells (Fig. 3A,B). Through biochemical assays, CCL2 enhanced activity of key glycolytic enzymes over time including: HK2, PKM1/2 and LDH, which was reduced by MET-KO (Fig. 3C–E). Reduction in enzymatic activity with MET-KO was associated with decreased protein levels of HK2 and LDH but not PKM1/2 (Fig. 3F). Overall, CCL2 facilitates glycolytic enzyme activity and expression through MET-dependent and independent mechanisms.

### Importance of MET in CCR2-mediated DCIS progression and metabolism

**Fig. 4:**
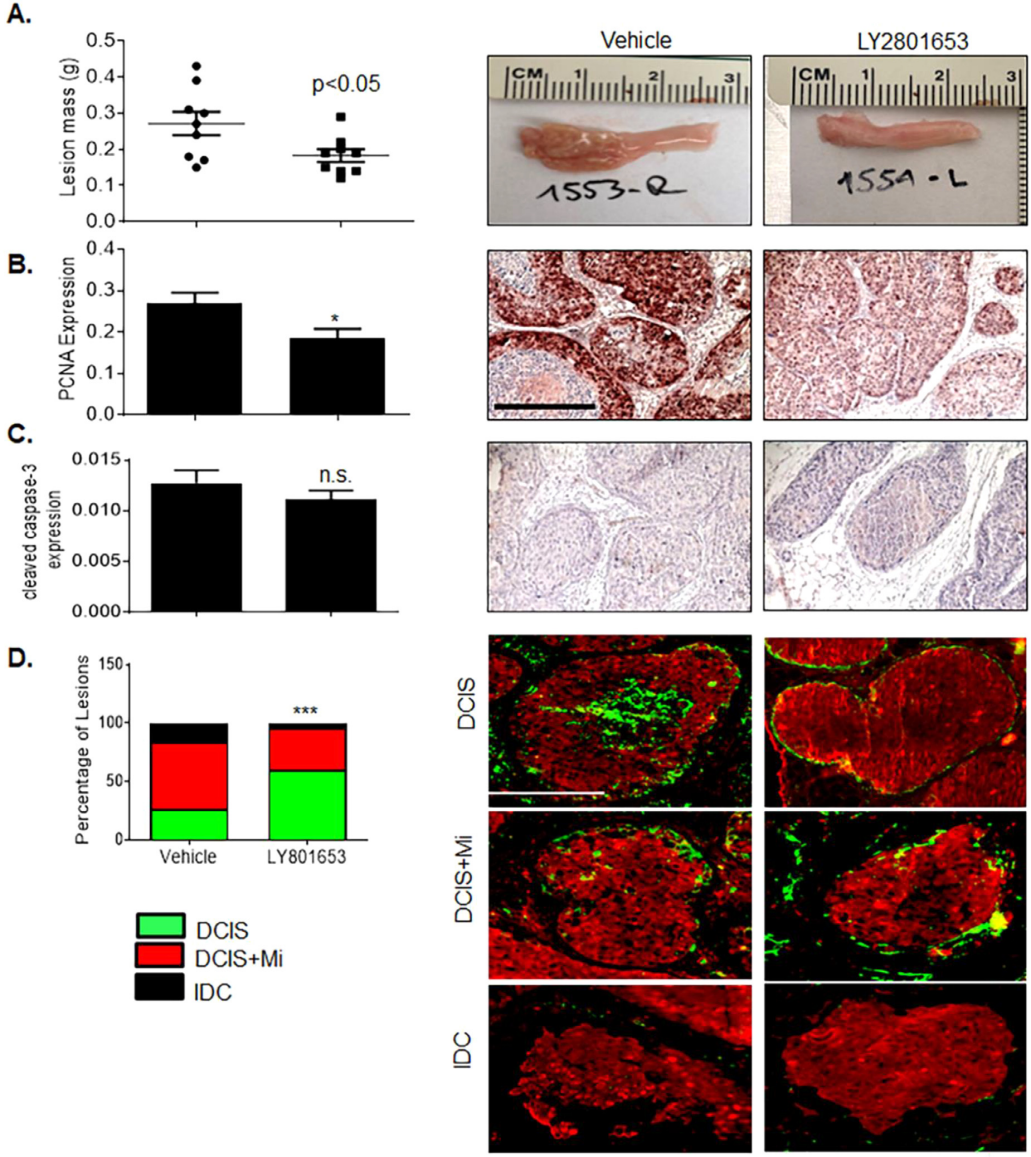
LY2801653 treatment inhibits growth and invasion of SUM225 CCR2-H MIND lesions. NOD- SCID mice bearing SUM225 CCR2-H MIND lesions were treated with 12 mg/kg LY2801653 or vehicle control for 4 weeks (*n = *8/group). Mammary tissues were measured for A. changes in mass, B. PCNA expression, C. cleaved caspase-3 expression or D. number of DCIS, DCIS+ microinvasive (DCIS+Mi) and invasive lesions through co-staining for a-sma (green) and CK/19 (red). Total number of lesions scored/group: *n = * 527 (vehicle), *n = * 948 (LY2801653). Statistical analysis was determined by Two Tailed t-test (A-C) or χ² test (D). Statistical significance was determined by *p < *0.05. *p < 0.05, ***p < 0.001, n.s=not significant. Mean+SEM are shown. Scale bar= 400 microns.

**Fig. 5:**
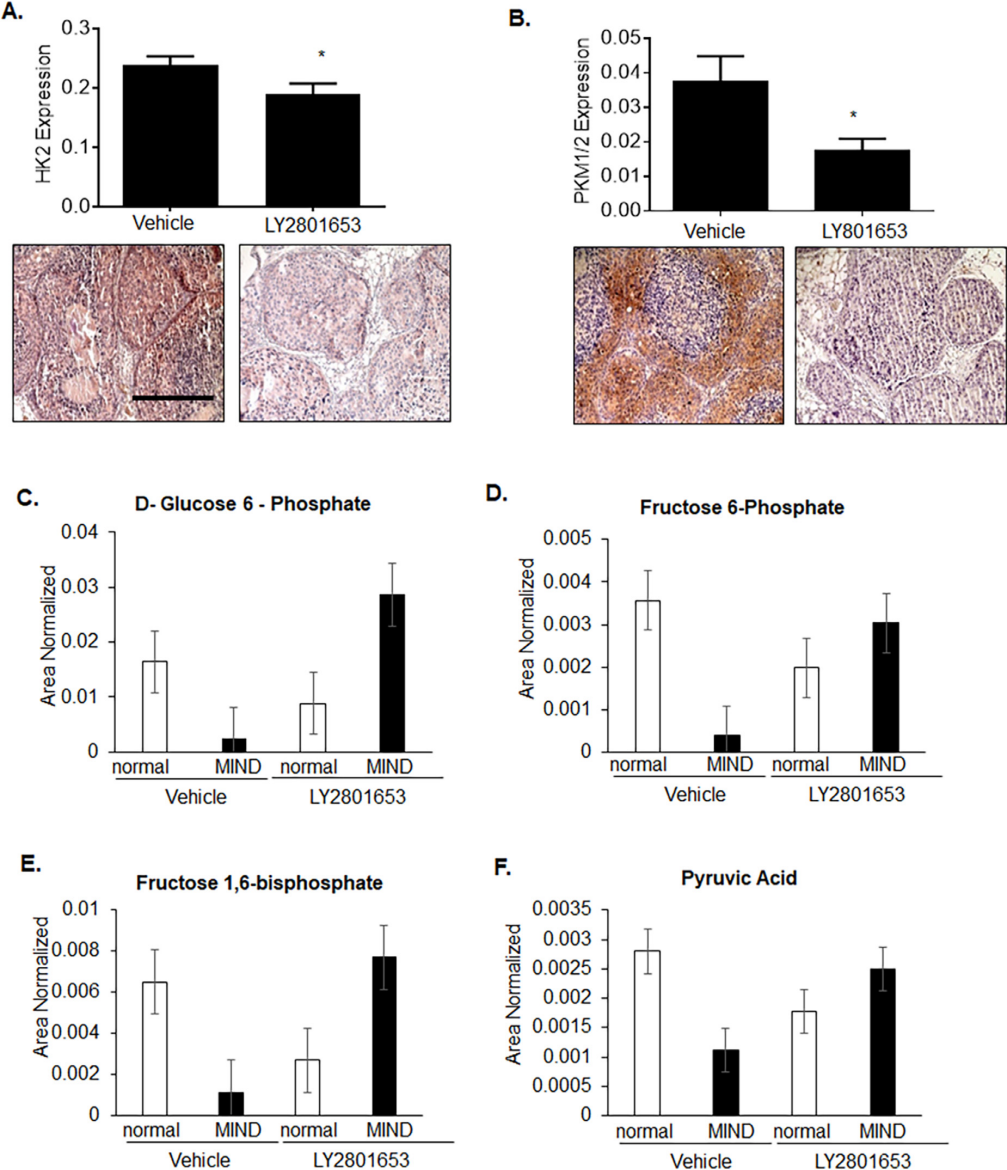
LY2801653 treatment affects glycolysis in SUM225 CCR2-H MIND lesions. A-B. SUM225 CCR2-H MIND lesions were treated with control vehicle or LY2801653 and immunostained for expression of HKII (A) or PKM1/2 (B). Scale bar= 400 microns. C-F. Normal mammary tissues or MIND lesions (*n = *3 per group) were subject to Ion chromatography-mass spectrometry analysis of glycolytic metabolites. Graphs were plotted as area normalized to tissue mass. The following glycolytic metabolites are shown: D-Glucose 6-phosphate (C), Fructose 6-phosphate (D), Fructose 1,6-bisphosphate (E) and Pyruvic Acid (F). Mean+SEM are shown.

We had previously shown that CCR2 overexpression in SUM225 breast carcinoma cells (CCR2-H) enhanced the growth and invasion of MIND lesions [15]. By immunohistochemistry, CCR2-H lesions showed increased MET phosphorylation (Supplemental Fig. 5). Using this model, we determined the effects of LY2801653 treatment to CCR2-mediated DCIS progression and metabolism. Compared to vehicle control (*n = *8), LY2801653 treatment reduced the growth of MIND lesions (Fig. 4A,B), but did not affect cell survival, as determined by immunostaining for PCNA and cleaved caspase-3 (Fig. 4C). LY2801653 resulted in fewer invasive carcinomas in mice; however more lesions were counted in this group (Fig. 4D). MIND lesions were assessed for changes in glycolytic enzyme expression and metabolites. Compared to vehicle control, LY2801653 reduced expression of HK2 and PKM1/2 and increased levels of glycolytic metabolites in MIND lesions, as determined by IC-MS (Fig. 5A–E). Overall, LY2801653 treatment reduced the growth and invasion and altered glucose metabolism of SUM225 CCR2-H MIND lesions.

To determine the associations between CCR2 and MET expression, we examined their

**Fig. 6:**
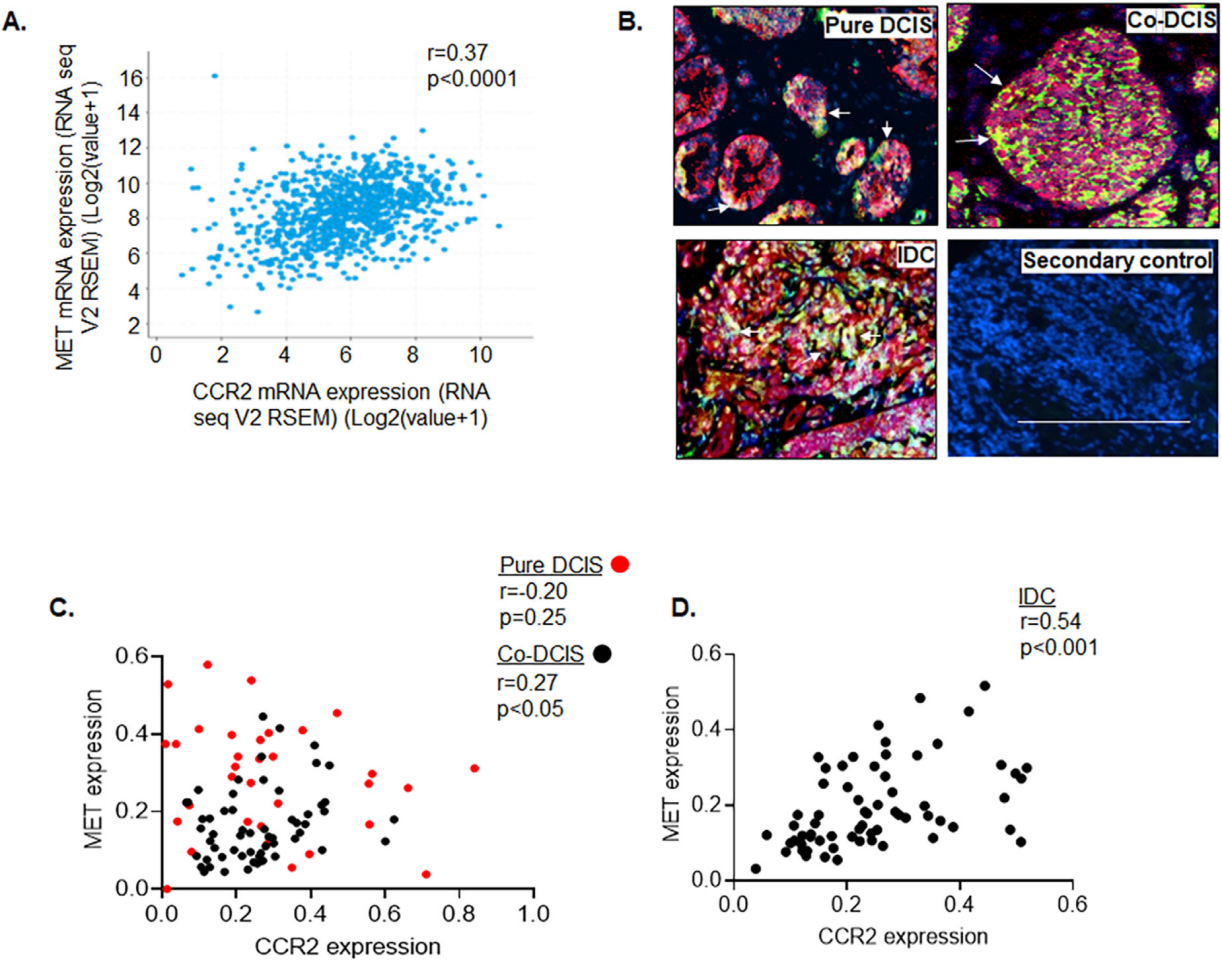
MET expression correlates to CCR2 expression in human breast carcinoma tissues. A. Spearman correlation test was performed for CCR2 and MET mRNA expression in IDC samples from TCGA datasets (cbioportal.org). *n = *963 B. Co-immunofluorescence staining was performed in normal breast, pure DCIS, DCIS co-occurring with IDC (co-DCIS) and IDC tissues for CCR2 (red) and MET (green) expression, with DAPI counterstain, *n = *9/group. Overlapping expression indicated by arrows. Scale bar=200 microns. C. MET and CCR2 expression in DCIS and IDC tissues were quantified by Image J and normalized to DAPI. C-D. CCR2 and MET protein expression were detected in pure DCIS (*n* = 33), Co-DCIS (*n = *58) (C) and IDC (D) (*n* = 67) tissues by DAB immunostaining. Expression was quantified by Image J. Statistical analysis was performed using Spearman correlation test. Statistical significance was determined by *p < *0.05.

To determine the associations between CCR2 and MET expression, we analyzed mRNA and protein levels in breast cancer. We detected significant associations between CCR2 and MET mRNA in IDC samples from TCGA datasets (Fig. 6A). Although DCIS is non-invasive cancer, DCIS co-occurring with IDC (co-DCIS) exhibit similar genetics to IDC, suggesting that co-DCIS may be more aggressive than pure DCIS [40]. Co-immunofluorescence staining of tissues revealed a greater overlap of CCR2 and MET expression in IDC and Co-DCIS compared to pure DCIS (Fig. 6B). Immunohistochemistry analysis revealed significant associations between MET and CCR2 protein expression in Co-DCIS and IDC but not in pure DCIS (Fig. 6C,D). Overall, these studies demonstrate a clinically relevant association between CCR2 and MET expression in DCIS and IDC tissues.

## Discussion

While studies have demonstrated an important role for CCL2/CCR2 signaling for DCIS progression, the mechanisms facilitating this process have remained unclear. Here, for the first time, we identify CCL2/CCR2 signaling interactions with MET receptors and demonstrate that MET is important in CCL2/CCR2-mediated DCIS progression and metabolism.

Interactions between GPCRs and RTKs have been identified in some studies but are largely not well understood [41]. For example, in neuronal cells, adenosine A_2A_ receptors complex with FGFR RTKs to modulate signaling, cellular differentiation and neuronal outgrowth [42]. Our studies indicate that MET is regulated through a novel CCL2/CCR2 interaction that involves SRC activation. Other SRC family members or adaptor proteins such as GRB2 might be involved in regulating CCR2/MET interactions [43,44]. Future studies would involve further characterizing the protein complexes involved in MET phosphorylation through proteomics analysis and identifying interacting binding sites among proteins through truncation and mutational analysis.

Our studies demonstrated that MET is important in CCL2/CCR2-mediated breast cancer growth and invasion. Interestingly, *in vivo* inhibition of MET reduced CCR2-mediated PKM protein expression but did not affect CCR2-mediated cell survival. *in vitro*, MET inhibition did not affect CCR2-mediated PKM expression but inhibited cell survival. It is not clear why we observed differences with *in vivo* and *in vitro* assays with MET inhibition. One possible reason may be due to signaling differences between CCR2 and MET in SUM225 with forced CCR2 overexpression, vs. DCIS.com and HCC1937 cell lines, which had endogenous CCR2 overexpression. While CCR2 overexpression in SUM225 cells enhanced MET expression, it may not have affected the same downstream pathways necessary for modulating cell survival and PKM expression in DCIS.com and HCC1937 cells. Another possible reason is hypoxia, which could have been enhanced with MET inhibition. HGF/MET signaling modulates HIF in tumor cells [45]. Hypoxia reduces activity of autophagic and pro-apoptotic pathways and modulate transcription of metabolic enzymes though HIF-dependent mechanisms [46]. Using cell-based models and *in vivo* tracing approaches, future studies would examine possible effects of hypoxia on metabolism in DCIS mediated by CCR2/MET signaling.

Interestingly, LY2801653 treatment in mice resulted in more lesions that were less invasive. The decreased invasiveness *in vivo* could be due partly to decreased cell migration as observed with MET-KO or LY2801653 treatment of cultured cells. We considered several potential reasons for the increased number of lesions. As LY2801653 treatment resulted in smaller lesions associated with decreased PCNA expression, the greater number of lesions were not likely due to increased cell proliferation. Furthermore, as inhibition of CCL2 signaling reduced the growth of breast tumor xenografts and decreased stem cell activity [28], cancer stem cell activity is not likely to be a factor. Rather, the greater number of lesions with LY2801653 treatment may be caused by slower DCIS progression. Decreased proliferation and migration could have resulted in smaller, more distinctive lesions, leading to a greater number detected by microscopy.

We predicted that MET inhibition would inhibit CCR2-mediated metabolism and DCIS progression. Interestingly, LY2801653 treatment led to accumulation of glycolytic metabolites and decreased invasiveness of MIND lesions. The increased metabolites could indicate enhanced glycolysis. However, it is more likely that LY2801653 treatment inhibited glycolysis in MIND lesions, as accumulation of glycolytic metabolites corresponded to decreased HK2 and PKM1/2 protein expression. The decreased expression of glycolytic enzymes could slow or delay the processing of metabolites, leading to their accumulation with MET inhibition. To further determine this possibility, it would be important to further characterize the effects of CCL2/CCR2 and MET on metabolic flux in breast cancer cells. Glutamine, a non-essential nutrient plays an important role in late-stage breast cancer [47]. Future studies would involve in-depth isotope tracing studies on breast cancer cell lines and tissues to determine the contributions of glucose and glutamine to CCL2/CCR2- and MET-mediated metabolism during DCIS progression.

Increased expression of glycolytic enzymes correlates with unfavorable prognosis for breast cancer patients [19]. Here, we show that CCL2 enhanced expression of glycolytic enzymes corresponding to increased enzymatic activity, which could help to confer a growth and invasive advantage to breast cancer cells. Interestingly, CCL2 enhanced HK2 protein levels as early as 15 min. HK2 is regulated at the transcriptional level by HIF1a, p53 or c-MYC [48]. It may also be regulated through protein stabilization [49]. CCR2-induction of enzyme protein levels represents one, but likely not the only mechanism for regulation of glycolysis in breast cancer cells. Other regulatory mechanisms for enzyme activity could include co-factors, binding of allosteric effectors such as ATP and covalent modifications such as phosphorylation [50]. Studies are being performed to further understand how CCL2/CCR2 signaling regulates metabolic enzyme expression and activity.

Here, we demonstrate that CCR2 and MET expression are associated with progressive disease. Interestingly, some pure DCIS cases showed associations between CCR2 and MET. However, we cannot draw conclusions about their potential invasiveness. To validate CCR2 and MET as a predictive marker for high-risk DCIS, it would be necessary to analyze a larger cohort of pure DCIS with follow-up data on recurrence and invasive disease.

## Conclusions

In summary, MET receptor activity is an important mechanism for CCL2/CCR2-mediated progression and metabolism of early-stage breast cancer, with important clinical implications.

## CRediT authorship contribution statement

**Diana Sofía Acevedo:** Investigation, Formal analysis, Visualization, Funding acquisition, Writing – original draft, Writing – review & editing. **Wei Bin Fang:** Investigation, Formal analysis. **Vinamratha Rao:** Investigation. **Vedha Penmetcha:** Formal analysis. **Hannah Leyva:** Investigation. **Gabriela Acosta:** Investigation. **Paige Cote:** Investigation. **Rebecca Brodine:** Investigation. **Russell Swerdlow:** Resources. **Lin Tan:** Formal analysis, Data curation. **Philip L Lorenzi:** Supervision. **Nikki Cheng:** Supervision, Conceptualization, Project administration, Funding acquisition, Writing – review & editing.

## Appendix Supplementary materials

Image, application 1
