# Supplementary material for: Regulation of growth, invasion and metabolism of breast ductal carcinoma through CCL2/CCR2 signaling interactions with MET receptor tyrosine kinases
Source: Neoplasia. 2022 Apr 8;28:100791. doi: 10.1016/j.neo.2022.100791 (PMC9010752; doi:10.1016/j.neo.2022.100791)
Supplement: Supplementary file 1 [file mmc1.pptx]

## Slide 1
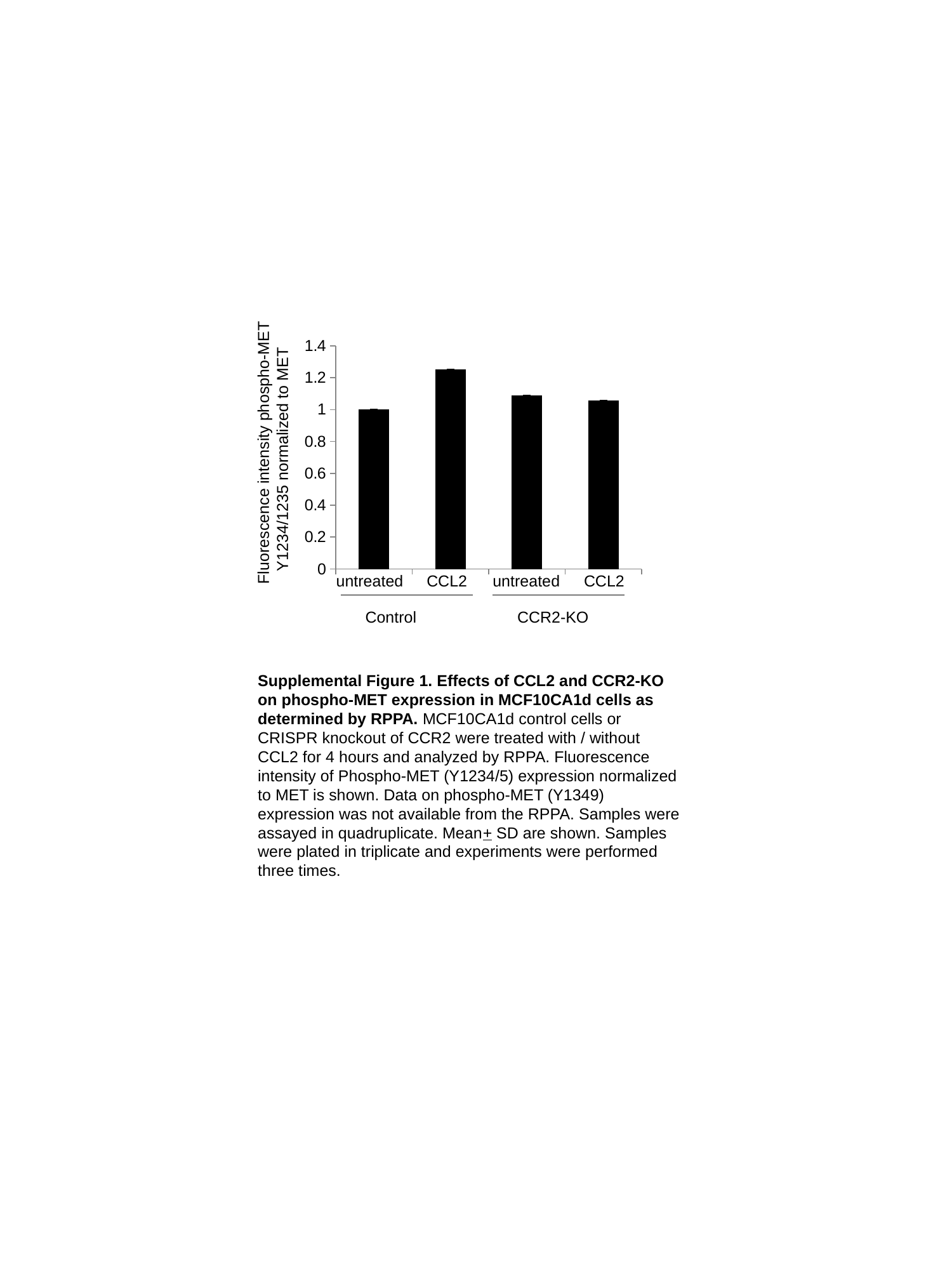

### Chart
| Category | |
|---|---| Fluorescence intensity phospho-MET Y1234/1235 normalized to MET
untreated
CCL2
untreated
CCL2
CCR2-KO
Control
Supplemental Figure 1. Effects of CCL2 and CCR2-KO on phospho-MET expression in MCF10CA1d cells as determined by RPPA. MCF10CA1d control cells or CRISPR knockout of CCR2 were treated with / without CCL2 for 4 hours and analyzed by RPPA. Fluorescence intensity of Phospho-MET (Y1234/5) expression normalized to MET is shown. Data on phospho-MET (Y1349) expression was not available from the RPPA. Samples were assayed in quadruplicate. Mean+ SD are shown. Samples were plated in triplicate and experiments were performed three times.

## Slide 2
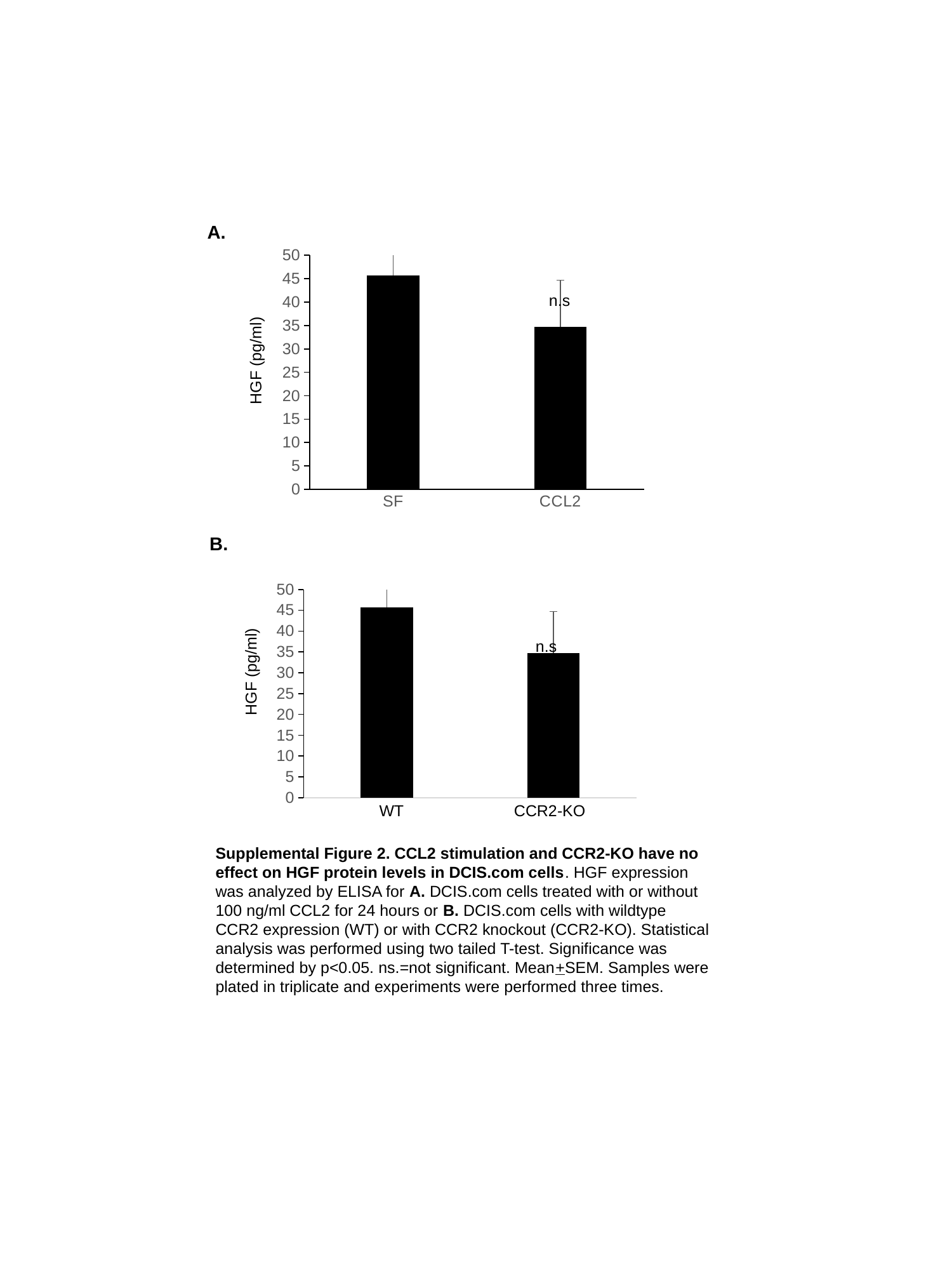

A.
### Chart
| Category | |
|---|---|
| SF | 45.74 |
| CCL2 | 34.66 |n.s
HGF (pg/ml)
B.
### Chart
| Category | |
|---|---|
| SF KO | 45.74 |
| CCL2 KO | 34.66 |n.s
HGF (pg/ml)
WT CCR2-KO
Supplemental Figure 2. CCL2 stimulation and CCR2-KO have no effect on HGF protein levels in DCIS.com cells. HGF expression was analyzed by ELISA for A. DCIS.com cells treated with or without 100 ng/ml CCL2 for 24 hours or B. DCIS.com cells with wildtype CCR2 expression (WT) or with CCR2 knockout (CCR2-KO). Statistical analysis was performed using two tailed T-test. Significance was determined by p<0.05. ns.=not significant. Mean+SEM. Samples were plated in triplicate and experiments were performed three times.

## Slide 3
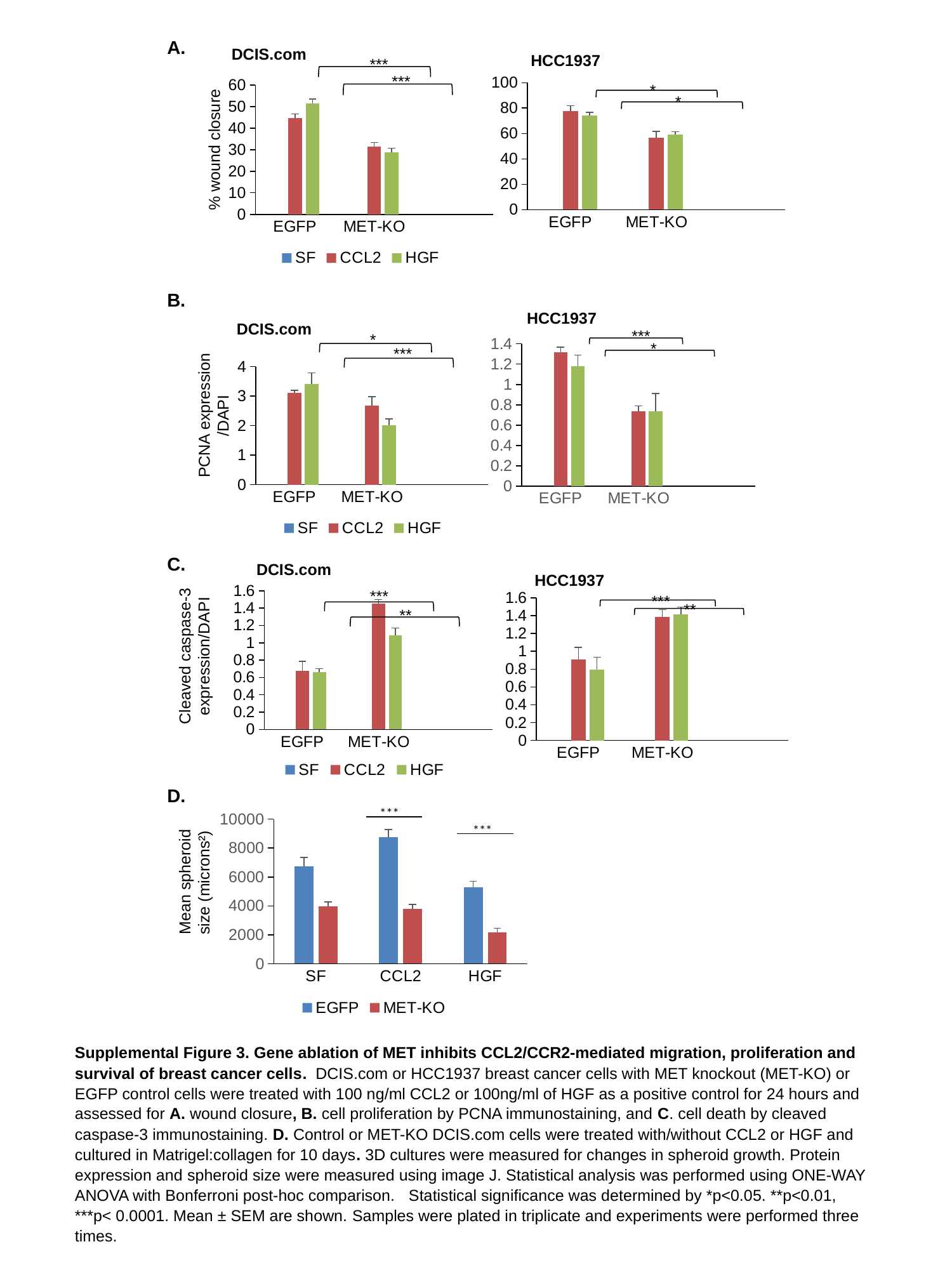

A.
DCIS.com
***
***
### Chart
| Category | SF | CCL2 | HGF |
|---|---|---|---|
| EGFP | 28.15 | 44.59 | 51.43 |
| MET-KO | 32.53 | 31.48 | 28.89 |% wound closure
HCC1937
### Chart
| Category | SF | CCL2 | HGF |
|---|---|---|---|
| EGFP | 60.89 | 77.67 | 74.33 |
| MET-KO | 61.31 | 56.7 | 59.24 |
*
*
B.
HCC1937
DCIS.com
*
***
### Chart
| Category | SF | CCL2 | HGF |
|---|---|---|---|
| EGFP | 1.708 | 3.103 | 3.416 |
| MET-KO | 1.249 | 2.684 | 2.011 |PCNA expression
/DAPI
***
### Chart
| Category | SF | CCL2 | HGF |
|---|---|---|---|
| EGFP | 0.9757 | 1.315 | 1.182 |
| MET-KO | 0.9076 | 0.7364 | 0.7349 |
*
C.
DCIS.com
### Chart
| Category | SF | CCL2 | HGF |
|---|---|---|---|
| EGFP | 1.199 | 0.6783 | 0.6578 |
| MET-KO | 1.53 | 1.45 | 1.085 |
***
**
Cleaved caspase-3 expression/DAPI
HCC1937
### Chart
| Category | SF | CCL2 | HGF |
|---|---|---|---|
| EGFP | 1.214 | 0.906 | 0.7937 |
| MET-KO | 1.576 | 1.387 | 1.417 |
***
**
D.
***
***
### Chart
| Category | EGFP | MET-KO |
|---|---|---|
| SF | 6738.0 | 3958.0 |
| CCL2 | 8748.0 | 3794.0 |
| HGF | 5274.0 | 2185.0 |Mean spheroid size (microns²)
Supplemental Figure 3. Gene ablation of MET inhibits CCL2/CCR2-mediated migration, proliferation and survival of breast cancer cells. DCIS.com or HCC1937 breast cancer cells with MET knockout (MET-KO) or EGFP control cells were treated with 100 ng/ml CCL2 or 100ng/ml of HGF as a positive control for 24 hours and assessed for A. wound closure, B. cell proliferation by PCNA immunostaining, and C. cell death by cleaved caspase-3 immunostaining. D. Control or MET-KO DCIS.com cells were treated with/without CCL2 or HGF and cultured in Matrigel:collagen for 10 days. 3D cultures were measured for changes in spheroid growth. Protein expression and spheroid size were measured using image J. Statistical analysis was performed using ONE-WAY ANOVA with Bonferroni post-hoc comparison.  Statistical significance was determined by *p<0.05. **p<0.01, ***p< 0.0001. Mean ± SEM are shown. Samples were plated in triplicate and experiments were performed three times.

## Slide 4
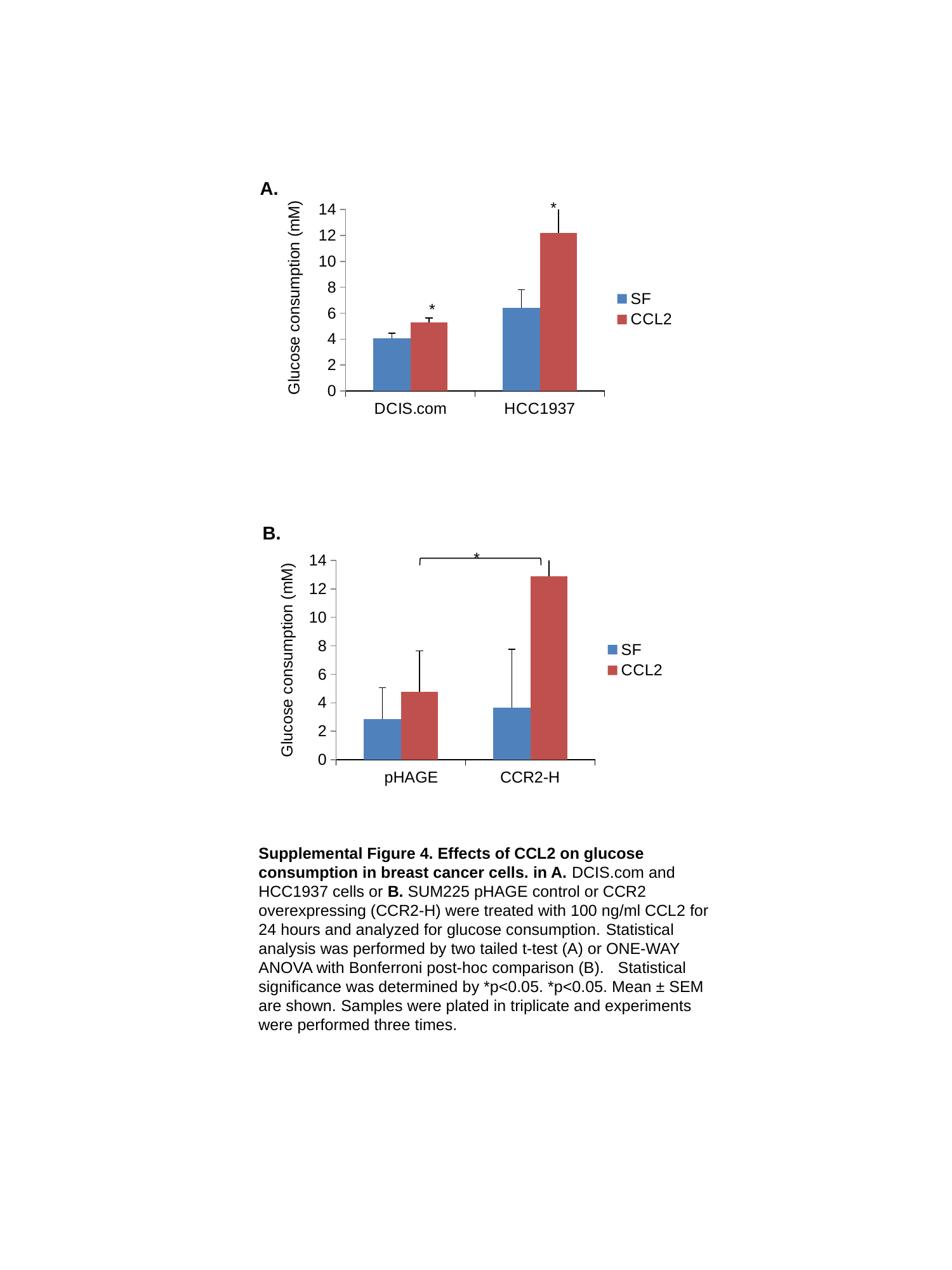

A.
*
### Chart
| Category | SF | CCL2 |
|---|---|---|
| DCIS.com | 4.057594529185816 | 5.273207664468572 |
| HCC1937 | 6.396310749999998 | 12.185845749999999 |Glucose consumption (mM)
*
B.
*
### Chart
| Category | SF | CCL2 |
|---|---|---|
| pHAGE | 2.8465259166666645 | 4.750156416666665 |
| CCR2+ | 3.6599384166666646 | 12.892002249999997 |Glucose consumption (mM)
pHAGE CCR2-H
Supplemental Figure 4. Effects of CCL2 on glucose consumption in breast cancer cells. in A. DCIS.com and HCC1937 cells or B. SUM225 pHAGE control or CCR2 overexpressing (CCR2-H) were treated with 100 ng/ml CCL2 for 24 hours and analyzed for glucose consumption. Statistical analysis was performed by two tailed t-test (A) or ONE-WAY ANOVA with Bonferroni post-hoc comparison (B).  Statistical significance was determined by *p<0.05. *p<0.05. Mean ± SEM are shown. Samples were plated in triplicate and experiments were performed three times.

## Slide 5
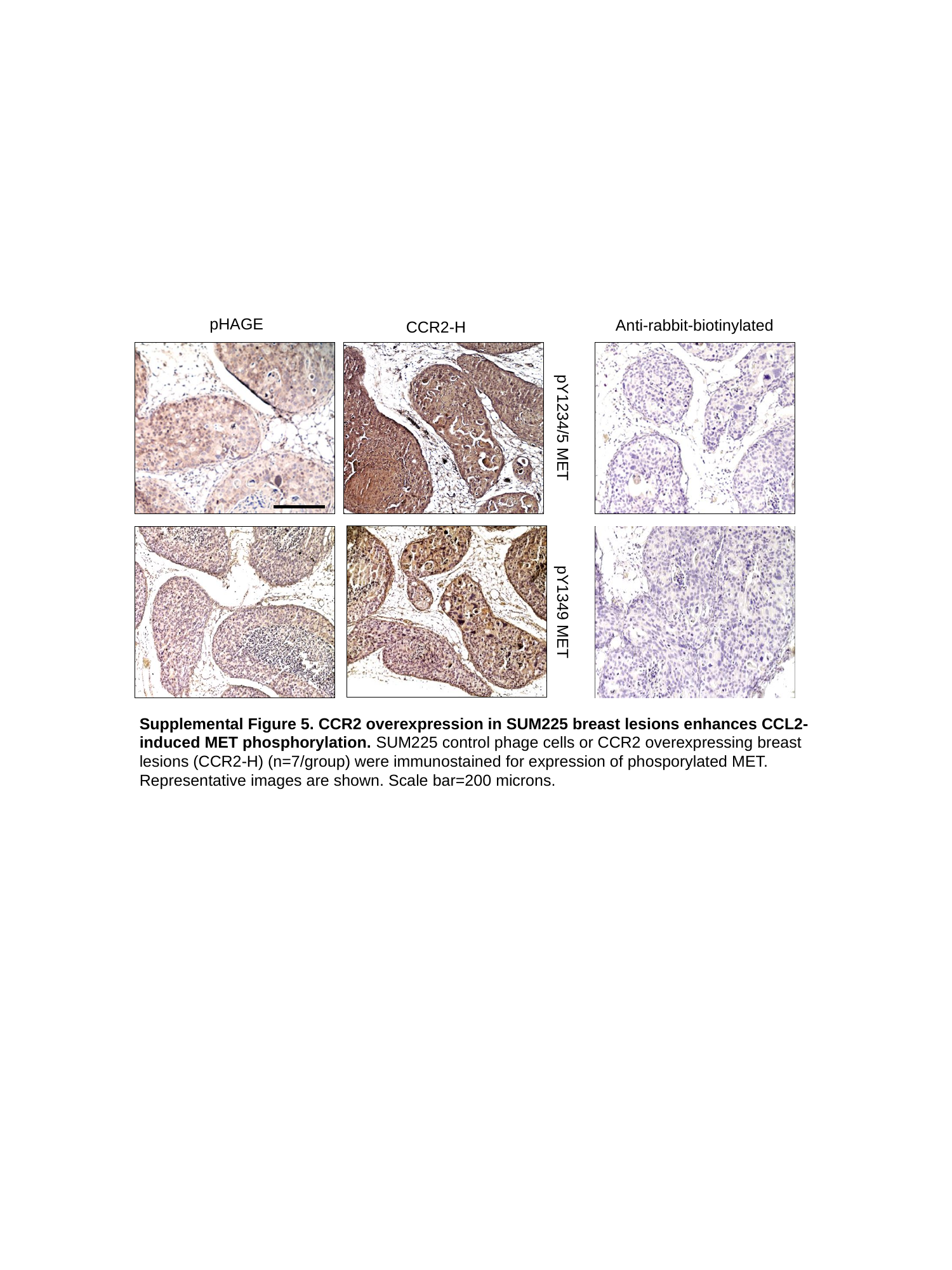

pHAGE
Anti-rabbit-biotinylated
CCR2-H
pY1234/5 MET
pY1349 MET
Supplemental Figure 5. CCR2 overexpression in SUM225 breast lesions enhances CCL2-induced MET phosphorylation. SUM225 control phage cells or CCR2 overexpressing breast lesions (CCR2-H) (n=7/group) were immunostained for expression of phosporylated MET. Representative images are shown. Scale bar=200 microns.
